# Brain Activation during Perception and Anticipation of Dyspnea in Chronic Obstructive Pulmonary Disease

**DOI:** 10.3389/fphys.2017.00617

**Published:** 2017-08-23

**Authors:** Roland W. Esser, Maria C. Stoeckel, Anne Kirsten, Henrik Watz, Karin Taube, Kirsten Lehmann, Helgo Magnussen, Christian Büchel, Andreas von Leupoldt

**Affiliations:** ^1^Department of Systems Neuroscience, University Medical Center Hamburg-Eppendorf Hamburg, Germany; ^2^Pulmonary Research Institute at LungClinic Grosshansdorf, Airway Research Center North, German Center for Lung Research Grosshansdorf, Germany; ^3^Atem-Reha GmbH Hamburg, Germany; ^4^Research Group Health Psychology, University of Leuven Leuven, Belgium

**Keywords:** COPD, pathophysiology, management, treatment, dyspnea, anxiety, quality of life

## Abstract

**Background:** Dyspnea is the impairing cardinal symptom in COPD, but the underlying brain mechanisms and their relationships to clinical patient characteristics are widely unknown. This study compared neural responses to the perception and anticipation of dyspnea between patients with stable moderate-to-severe COPD and healthy controls. Moreover, associations between COPD-specific brain activation and clinical patient characteristics were examined.

**Methods:** During functional magnetic resonance imaging, dyspnea was induced in patients with stable moderate-to-severe COPD (*n* = 17) and healthy control subjects (*n* = 21) by resistive-loaded breathing. Blocks of severe and mild dyspnea were alternating, with each block being preceded by visually cued anticipation phases.

**Results:** During the perception of increased dyspnea, both patients and controls showed comparable brain activation in common dyspnea-relevant sensorimotor and cortico-limbic brain regions. During the anticipation of increased dyspnea, patients showed higher activation in hippocampus and amygdala than controls which was significantly correlated with reduced exercise capacity, reduced health-related quality of life, and higher levels of dyspnea and anxiety.

**Conclusions:** This study suggests that patients with stable moderate-to-severe COPD show higher activation in emotion-related brain areas than healthy controls during the anticipation, but not during the actual perception of experimentally induced dyspnea. These brain activations were related to important clinical characteristics and might contribute to an unfavorable course of the disease via maladaptive psychological and behavioral mechanisms.

## Introduction

Chronic Obstructive Pulmonary Disease (COPD) is a prevalent and debilitating respiratory disease, characterized by persistent and usually progressive airflow limitation (O'Donnell et al., 2007; GOLD, 2017). Dyspnea is the cardinal respiratory symptom of COPD, causing significant reductions in patients' exercise capacity and quality of life, and is frequently linked to comorbid anxiety and depression (Schlecht et al., 2005; Martínez Francés et al., 2008; Maurer et al., 2008; Roche, 2009; Yohannes and Alexopoulos, 2014; Waschki et al., 2015; GOLD, 2017). Dyspnea is usually experienced as highly aversive and threatening (American Thoracic Society, 1999; Parshall et al., 2012). Consequently, many patients avoid situations associated with dyspnea, especially physical activity (O'Donnell, 2006). This maladaptive avoidance behavior results in progressive deconditioning, ultimately increasing dyspnea at lower activity levels and contributing to disease progression (Reardon et al., 2006; Troosters et al., 2013; Waschki et al., 2015). In particular, the fearful anticipation of dyspnea is suggested to play a key role within this spiral of decline (Hayen et al., 2013).

The brain mechanisms underlying the perception and anticipation of dyspnea are still poorly understood. Previous neuroimaging studies in healthy volunteers have identified multiple dyspnea-related brain areas, presumably related to different aspects of dyspnea. Somatosensory and motor aspects are thought to be processed by sensorimotor areas (e.g., SM1, SII), while affective-motivational aspects are processed by cortico-limbic areas including anterior insula, anterior cingulate cortex (ACC), hippocampus, prefrontal cortex (PFC), and amygdala (von Leupoldt and Dahme, 2005; Davenport and Vovk, 2009; Evans, 2010). Preliminary findings suggest an involvement of several of these areas such as the insula, amygdala, ACC and the periaqueductal gray (PAG) during the fearful anticipation of impending dyspnea (Stoeckel et al., 2015, 2016; Faull et al., 2016). This converges with studies on other aversive states, e.g., fear, aversive learning, and pain, reporting activation in similar cortico-limbic structures (Peyron et al., 2000; Apkarian et al., 2005; Sehlmeyer et al., 2009; Wiech and Tracey, 2013). Unfortunately, neuroimaging studies with high spatial resolution (e.g., functional magnetic resonance imaging [fMRI]) on the anticipation and perception of dyspnea in COPD patients are widely absent, which greatly limits our understanding of the potential contribution of disease-specific brain mechanisms to the patients' burden.

However, COPD-specific brain mechanisms due to chronic dyspnea experiences seem highly plausible (Herigstad et al., 2011). For example, contemporary models on the effects of chronic pain suggest that brain activation in pain patients shifts with chronicity from sensory to emotion-related areas, leading to increased activation preferentially in cortico-limbic structures (Apkarian et al., 2011; Mansour et al., 2014). Moreover, enhanced activation was recently demonstrated in emotion-related brain areas (i.e., PFC, ACC) of COPD patients while reading dyspnea-related statements (Herigstad et al., 2015).

This fMRI-study aimed (1) to compare brain activation during the perception and anticipation of resistive-load induced increased dyspnea between patients with COPD and healthy control subjects and (2) to evaluate potential associations with patient characteristics. Analogous to findings on chronic pain (Apkarian et al., 2005, 2011; Mansour et al., 2014; Baliki and Apkarian, 2015), we hypothesized that during dyspnea perception, both groups activate similar dyspnea-relevant brain areas, with patients showing higher activation in cortico-limbic structures. Based on findings on fear, aversive learning, and dyspnea anticipation (Sehlmeyer et al., 2009; Wiech and Tracey, 2013; Stoeckel et al., 2015; Faull et al., 2016), we further hypothesized that patients show enhanced activation in emotion-related brain areas during dyspnea anticipation.

## Methods

### Participants

Seventeen outpatients with stable (no exacerbation within the last year) moderate-to-severe COPD (GOLD stage 2 and 3) were recruited at a Pulmonary Research Institute at LungClinic Grosshansdorf, Grosshansdorf, Germany (14 patients) and an outpatient pulmonary rehabilitation center (three patients before start of the rehabilitation program; Atem-Reha GmbH, Hamburg, Germany). The control group consisted of 21 individuals, recruited from the database of the Pulmonary Research Institute and was matched for age, sex, and body-mass-index. All subjects participated in a previous study on structural brain changes in COPD (Esser et al., 2016). This study was approved by the ethics committees of the Medical Associations Hamburg (PV3006) and Schleswig-Holstein (IV/EK/122/08), and was conducted according to the Declaration of Helsinki. Written informed consent was obtained from each participant prior to testing.

Post-bronchodilator lung function (forced expiratory volume in 1 s, FEV_1_; forced vital capacity, FVC) was measured using standard spirometry (Miller et al., 2005), based on established reference values (Quanjer et al., 1993). All control subjects had normal lung function (FEV_1_%pred > 80%; FEV1/FVC > 0.7) and no history of respiratory disease. All participants were screened using a standardized diagnostic interview to exclude psychiatric or neurological disorders (First et al., 1996). Furthermore, participants completed the Hospital Anxiety and Depression Scale (HADS) to assess individual levels of anxiety and depression (Herrmann et al., 1995). In patients, further clinical characteristics were collected including exercise capacity (6-min-walk distance, 6MWD) (ATS Committee on Proficiency Standards for Clinical Pulmonary Function Laboratories, 2002), disease-specific quality of life (St. George's Respiratory Questionnaire, SGRQ) (Jones et al., 1992), and degree of dyspnea during daily activities (modified Medical Research Council Dyspnea Scale, mMRC) (Bestall et al., 1999).

### Apparatus and respiratory parameters

Participants breathed through a face mask, connected to an MR-compatible pneumotachograph (ZAN 600 unit, ZAN Messgeräte GmbH, Oberhulba, Germany) ending in a two-way non-rebreathing valve. A 2.6 m tube (diameter: 3.5 cm) was permanently connected to the inspiratory port of the valve, while the expiratory port was left free. This enabled the easy introduction and removal of MR-compatible, flow-resistive loads of variable magnitudes at the distal end of the tube. These loads (in-house manufactured) consisted of MR-compatible resistive screens of different porosity, which are permanently mounted in hard plastic casings. Each of these loads provides a known, calibrated resistance to the inspiratory air flow during loaded conditions, which is depending on the porosity of the screens (i.e., the lower the porosity, the higher the resistance). Respiratory parameters including partial pressure of end-tidal CO_2_ (PET_CO2_), peak inspiratory pressure (P_I_), tidal volume (V_T_), breathing frequency (f), minute ventilation (V_E_), and inspiratory time (T_I_) were measured continuously.

### Experimental protocol

One day prior to MRI testing, each subject was placed in a supine position (outside the scanner) and presented with different inspiratory loads of increasing magnitude. Each load was presented for 24 s and subsequently rated with regard to dyspnea intensity using a Borg Scale (0 = “not noticeable” to 10 = “maximally imaginable”). The sensation of dyspnea was explained as “uncomfortable or difficult breathing” to our participants. Load magnitude was increased until subjects reliably reported a sensation of “severe” dyspnea (Borg Score ≥ 5). That load was then used to induce severe dyspnea during scanning. For the mild dyspnea condition, the smallest load that was reliably rated as different from unloaded breathing was used.

On the day of MRI scanning and after completing the questionnaires, participants were familiarized with the experimental design and set-up to avoid unintended learning processes during MRI data acquisition. Therefore, each subject underwent a computer-based standardized instruction, including explicit details on the associations between visual cues and experimental conditions, outside the scanner. Participants learned the association between different colors of the fixation cross indicating “mild” and “severe” load condition and changes in the thickness of fixation crosses used to differentiate between unloaded anticipation (thin cross) and loaded breathing (thick cross) periods. Furthermore, subjects were acquainted with the button response system used for Borg Scale ratings. Then, participants were connected to the breathing circuit and entered the scanner. Visual cues and Borg Scales were projected into the scanner bore via a mirror system, using Presentation software (Neurobehavioral Systems, Inc., Albany, CA). A test run allowed subjects to familiarize themselves with the button-box response system and all cues and scales.

**Figure 1:**
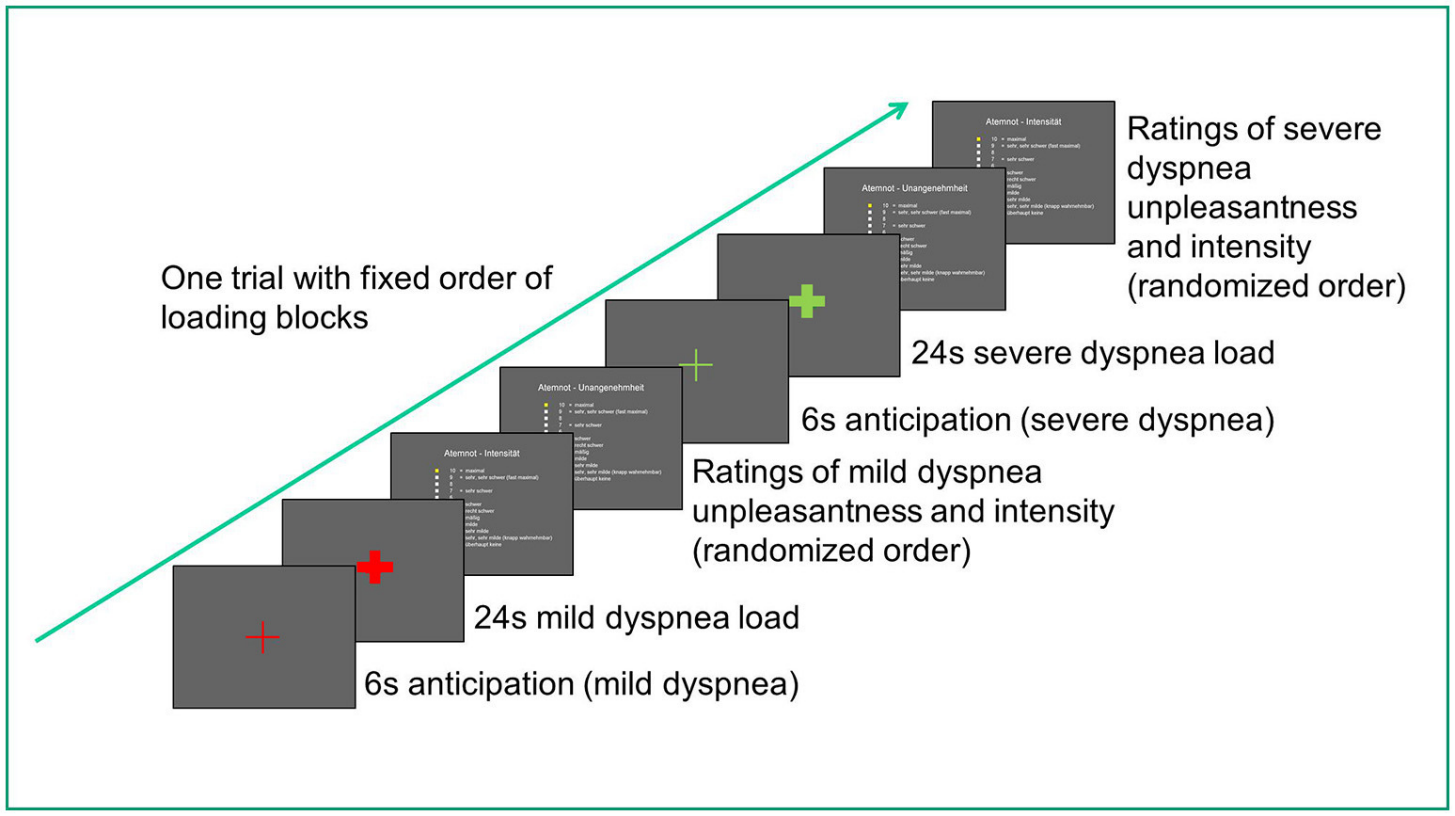
Diagram of scanning protocol for one trial (out of ten). The entire scan duration was about 13–17 min and differed between participants due to differences in individual rating speed.

During MRI scanning, 10 blocks of mild dyspnea and 10 blocks of severe dyspnea were presented, each lasting 24 s. In a fixed order, each mild dyspnea condition was followed by a severe dyspnea condition. Each loaded dyspnea condition was preceded by a 6 s visually cued, unloaded anticipation period signaling with 100% contingency the upcoming load condition. Each loaded condition was followed by Borg Scale ratings of dyspnea intensity and unpleasantness, presented in randomized order. Borg Scale ratings lasted approximately 20 s and served to recover from loaded breathing as well as to re-establish baseline breathing before the next load condition. All experimental events and Borg Scale ratings were recorded via the Presentation software (for a diagram of scanning protocol, see Figure 1).

### Data analysis

Respiratory parameters and Borg Scale ratings were averaged across respective experimental conditions and analyzed with SPSS Statistics 22 software (SPSS Inc., Chicago, IL), using a significance level of *p* < 0.05. We employed two-sample *t*-tests, Mann-Whitney-*U*-tests (non-normal distributions), and χ^2^-tests (frequency data).

MRI data were obtained on a 3T Magnetom-TRIO System (Siemens, Erlangen, Germany) using a 32-channel head coil. Functional MRI scans were acquired with an echo planar imaging ${\text{T}}_{2}^{*}$-sensitive sequence (48 contiguous axial slices in descending order, 2 mm slice thickness with 1 mm gap, *TR* = 2870 ms, *TE* = 25 ms, flip angle = 80°, field of view = 208 × 208 mm). After the fMRI-measurement, we collected a high-resolution T1-weighted structural brain image (MP-RAGE sequence, 1 mm isotropic voxel size, 240 slices). fMRI data preprocessing and statistical analysis was carried out using SPM8 (Statistical Parametric Mapping, http://www.fil.ion.ucl.ac.uk/spm) within Matlab2013a (The MathWorks, Inc., Natick, Massachusetts, United States). The first two blocks of each condition were excluded from the analysis to guarantee full adaptation to scanner environment and experimental conditions. All images were unwarped and realigned to the first image, coregistered to the individual high resolution T1 structural image, normalized to the standard template, and finally smoothed with an isotropic full-width at half-maximum Gaussian kernel of 8 mm. Severe dyspnea conditions were contrasted with mild dyspnea conditions rather than resting baseline conditions in order to guarantee that attention is similarly focused to the respiratory sensations in both contrasted conditions and not free-floating as in rest conditions, which could have biased brain activation patterns. Additionally, some individuals might have experienced rest conditions as relieving compared to the more aversive character of the other conditions, which could have resulted in confounding relief-related brain activations instead of non-stimulus resting state activation patterns.

On single-subject level, the statistical model included regressors for cue mild dyspnea, mild dyspnea, cue severe dyspnea, severe dyspnea, and ratings. Mean global signal intensity of each volume and PET_CO2_ time-logged to the beginning of each scan were included as covariates-of-no-interest, to correct for a potential effect of respiratory fluctuations on brain response. To compare individual results on group-level, contrasts for the perception of increased dyspnea [severe vs. mild dyspnea] and for the anticipation of increased dyspnea [cue mild vs. cue severe dyspnea] were created for each participant to calculate separate one-sample *t*-tests for the patient and control group. A conjunction null analysis tested for brain areas showing shared significant activation between both groups. Group differences in neural activation for the perception and anticipation of increased dyspnea were tested with two-sample *t*-tests. Here, smoking status (i.e., current, former, and never smoker) was included as covariate-of-no-interest.

Differences in neural activation were accepted as significant if exceeding a threshold of *p* < 0.05 after whole-brain family-wise error correction (FWE) or family-wise error correction within predefined regions-of-interest (ROI). ROIs were based on previous results on the neural processing of dyspnea (Davenport and Vovk, 2009; Evans, 2010; Herigstad et al., 2011), pain (Peyron et al., 2000; Apkarian et al., 2005), fear and aversive learning (Sehlmeyer et al., 2009; Wiech and Tracey, 2013), and included primary sensorimotor cortex (SM1), secondary somatosensory cortex (SII)/operculum, supplementary motor area (SMA), insula, ACC, thalamus, PFC, hippocampus, amygdala, and midbrain/PAG. Bilateral masks were generated from the automated anatomical labeling (AAL) template (Tzourio-Mazoyer et al., 2002). For display purposes, we used a threshold of p uncorrected < 0.001 for all figures.

Associations of brain activation with patient characteristics (disease duration; exercise capacity, 6MWD; quality of life, SGRQ; level of dyspnea, mMRC; and HADS anxiety and depression scores) were examined by off-line partial correlation analyses (controlled for smoking status).

## Results

### Participants

As expected, patients showed significantly lower lung function and a greater proportion of current and former smokers compared to controls. There were no significant group differences regarding sex, age, height, weight, body-mass-index, and HADS scores. Furthermore, none of the participants showed clinically relevant HADS depression or anxiety scores. Patients with COPD had a relatively preserved 6MWD and minor exertional dyspnea according to the mMRC grades (Table 1).

**Table 1:** Baseline characteristics.

	**COPD**	**Controls**
Subjects, n	17	21
Females/males, n	8/9	11/10
Age, yr.	65.6 (9.3)	63.4 (8.8)
Height, cm	171.0 (8.2)	173.4 (9.4)
Weight, kg	75.1 (10.7)	77.5 (13.0)
Body mass index, kg/m^2^	25.8 (3.9)	25.7 (3.3)
FEV_1_, % predicted	47.2 (10.9)	122.9 (10.3)^***^
FVC, % predicted	93.1 (12.4)	126.2 (13.0)^***^
FEV_1_/FVC, %	41.0 (9.0)	79.8 (4.7)^***^
**SMOKING STATUS, %**		
- Current smoker	53%	10%^***^
- Former smoker	47%	19%^***^
- Never smoker	–	71%
Depression (HADS)	3.2 (2.0)	2.0 (2.2)
Anxiety (HADS)	3.1 (2.2)	2.1 (2.2)
**DISEASE SEVERITY, n**		
- Moderate (GOLD stage II)	4	–
- Severe (GOLD stage III)	13	–
Disease duration, years	11.0 (6.2)	–
Exercise capacity (6MWD), m	490 (70)	–
Quality of life (SGRQ)	37.6 (13.5)	–
Level of dyspnea (mMRC)	1.06 (0.8)	–

*Abbreviations: FEV_1_, forced expiratory volume in 1s; FVC, forced vital capacity; HADS, Hospital Anxiety and Depression Scale; 6MWD, 6-min-walk distance; SGRQ, St. George's Respiratory Questionnaire; mMRC, modified Medical Research Council Dyspnea Scale*.

*Data are presented as mean (SD)*.

*** *p < 0.001 for the comparison between COPD and control group*.

### Dyspnea ratings

Borg Scale ratings confirmed successful induction of mild and severe dyspnea. Ratings for dyspnea intensity and unpleasantness were significantly higher for the severe compared to the mild dyspnea condition without significant differences between groups. Although patients reported slightly higher intensity and unpleasantness of mild dyspnea (most likely reflecting the chronic respiratory impairments in these patients), the increases from mild to severe dyspnea conditions (i.e., Δ intensity and Δ unpleasantness) were comparable between groups (Table 2).

**Table 2:** Resistive load magnitudes and Borg Scale dyspnea ratings.

	**COPD**	**Controls**
**MILD DYSPNEA**		
- Load magnitudes, kPa/l/s	0.2 (0.2)	0.3 (0.2)
- Intensity ratings	1.7 (1.1)	0.9 (0.9)^*^
- Unpleasantness ratings	1.9 (1.4)	0.9 (0.8)^**^
**SEVERE DYSPNEA**		
- Load magnitudes, kPa/l/s	2.0 (1.0)	3.3 (1.6)^**^
- Intensity ratings	5.1 (1.8)	4.5 (2.4)
- Unpleasantness ratings	4.9 (2.1)	4.3 (2.5)
Δ Intensity	3.4 (1.7)	3.6 (1.8)
Δ Unpleasantness	3.0 (1.6)	3.4 (2.0)

*Data are presented as mean (SD). Δ intensity and Δ unpleasantness ratings represent the difference of ratings for the severe dyspnea condition minus the mild dyspnea condition*.

* *p < 0.05*,

** *p < 0.01 for the comparison between COPD and control group*.

### Respiratory parameters

Both groups showed comparable, mildly hypocapnic breathing patterns during the perception (severe vs. mild dyspnea) and anticipation (cue severe vs. cue mild dyspnea) of dyspnea (Supplementary Data Sheet: Table S1). Although patients compared to controls demonstrated different absolute values of breathing frequency, minute ventilation, and inspiratory time throughout experimental conditions, the differences in respiratory parameters (i.e., Δ values; Table 3) were comparable between groups, suggesting comparable respiratory changes in both patients and controls (see Supplementary Data Sheet, for more detailed information on respiratory parameters).

**Table 3:** Group means (SD) for Δ respiratory parameters of dyspnea anticipation (cue severe dyspnea minus cue mild dyspnea) and dyspnea perception (severe dyspnea minus mild dyspnea) for patients with COPD and control subjects.

	Δ **anticipation of dyspnea**		Δ **perception of dyspnea**	
	**COPD**	**Controls**	**COPD**	**Controls**
PET_CO2_, mmHg	−0.19 (0.92)	−0.45 (0.95)	−0.27 (0.7)	0.07 (0.56)
P_I_, mbar	−0.29 (0.92)	0.18 (1.51)	7.48 (4.39)	6.09 (3.37)
V_T_, L	−0.03 (0.15)	−0.03 (0.21)	−0.1 (0.13)	−0.13 (0.26)
f, breaths/min	−0.37 (2.07)	0.23 (1.95)	0.13 (1.49)	−0.59 (1.46)
V_E_, L/min	−1.1 (0.99)	−0.66 (2.56)	−1.71 (1.1)	−2.55 (2.55)
T_I_, s	0.02 (0.26)	−0.13 (0.58)	0.19 (0.21)	0.27 (0.48)

*Abbreviations: PET_CO2_, partial pressure of end-tidal CO_2_; P_I_, Peak inspiratory pressure; V_T_, Tidal volume; f, Breathing frequency; V_E_, Minute ventilation; T_I_, Inspiratory time*.

### Functional imaging data

#### Perception of dyspnea

**Figure 2:**
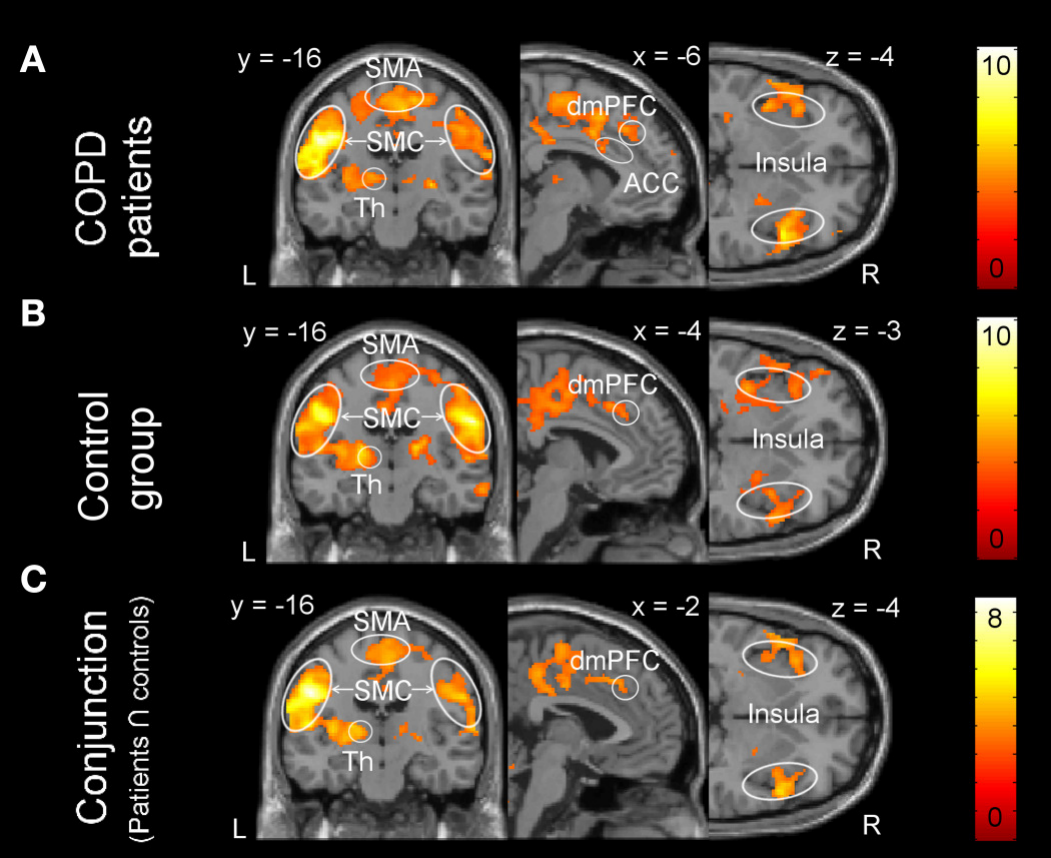
Brain activation during the perception of increased dyspnea. **(A)** Patients with COPD showed significant activation in the supplementary-motor area (SMA), sensorimotor cortices (SMC: primary sensorimotor cortex and secondary somatosensory cortex/operculum), thalamus (Th), anterior cingulate cortex (ACC), dorso-medial prefrontal cortex (dmPFC), and insula. **(B)** The control group showed significant activation in comparable brain areas. **(C)** The conjunction analysis (patients ∩ control subjects) revealed shared brain activation during increased dyspnea perception between the patient and control group. For visual purposes, activation is thresholded at p_uncorrected_ < 0.001 with colorbars indicating *T*-values.

One-sample within group *t*-tests for [severe vs. mild dyspnea] showed a widely comparable pattern of brain activation in both groups during the perception of increased dyspnea (Figures 2A,B). This included areas related to sensorimotor processes (i.e., SM1, SII/operculum, SMA, and thalamus) and related to emotional/cognitive processing, such as insula, ACC, and PFC (Table 4 for patient group; see Supplementary Data Sheet for control group: Table S2). The conjunction analysis confirmed these findings by demonstrating a massive overlap of neural activation patterns between the patient and control group (Figure 2C, and Supplementary Data Sheet: Table S3). Notably, two-sample *t*-tests revealed no group differences in brain activation during increased dyspnea perception, i.e., neither enhanced nor reduced activation was observed in patients as compared to control subjects.

**Table 4:** MNI-space peak coordinates, *z*-values, and *p*-values for regions showing significant brain activation during increased dyspnea perception in patients with COPD.

**Brain Region**	**x**	**y**	**z**	**Z**	***P***
L SM1	−54	−18	34	5.58	0.003^†^
R	66	−2	14	4.70	0.008^*^
L SII/operculum	−58	2	12	5.14	0.019^†^
R	64	6	12	4.89	0.001^*^
R SMA	10	6	46	4.78	0.002^*^
L Thalamus	−14	−22	−6	3.84	0.025^*^
**L INSULAR CORTEX**					
- Posterior	−40	−4	−2	4.64	0.003^*^
- Anterior	−38	20	2	3.99	0.030^*^
R	50	10	−6	4.97	0.038^†^
L ACC	−6	14	28	3.74	0.045^*^
**R PFC**					
- Dorso-medial	4	20	42	4.40	0.009^*^
- Lateral	24	48	28	4.98	0.036^†^
L Cerebellum	−14	−62	−22	4.95	0.040^†^

*Abbreviations: L, left hemisphere; R, right hemisphere; SM1, primary sensorimotor cortex; SII, secondary somatosensory cortex; SMA, supplementary-motor area; ACC, anterior cingulate cortex; PFC, prefrontal cortex*.

† *whole-brain family-wise error corrected*,

* *corrected for multiple comparisons within respective bilateral ROIs*.

#### Anticipation of dyspnea

**Figure 3:**
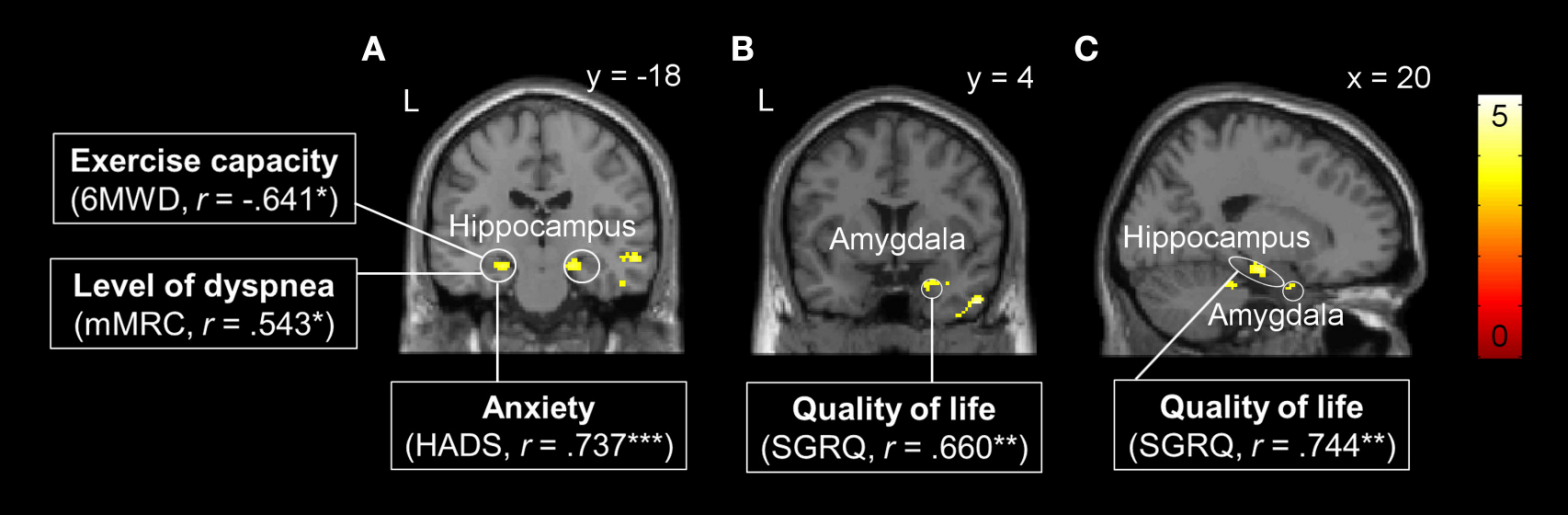
Brain areas with significantly higher neural activation in patients with COPD compared to the control group during the anticipation of increased dyspnea. **(A)** Bilateral hippocampus, **(B)** right amygdala, and **(C)** right hippocampus and amygdala. Enhanced neural activation in these brain regions was correlated with patient characteristics such as reduced exercise capacity, higher level of dyspnea, and anxiety in the left hippocampus **(A)**, and reduced quality of life in right hippocampus **(B)** and right amygdala **(C)**. For visual purposes, activation is thresholded at *p*_uncorrected_ < 0.001 with colorbars indicating *T*-values. Significant correlations are presented as ^*^p < 0.05, ^**^p < 0.01, ^***^p < 0.001.

One-sample *t*-tests for [cue severe vs. cue mild dyspnea] showed no statistically significant brain activation in any of the two groups, neither in the whole-brain nor in the ROI-based approach. However, two-sample *t*-tests revealed significantly higher neural activation in the right amygdala (peak: x, y, z = 36, 2, −24, Z = 3.33, ROI-corrected *p* = 0.025) and bilateral hippocampus (left peak: x, y, z = −24, −14, −14, Z = 4.38, ROI-corrected *p* = 0.002; right peak: x, y, z = 20, −16, −16, Z = 3.82, ROI-corrected *p* = 0.019; Figure 3) in the patient group compared to control subjects. There were no differences between groups at a whole-brain level and no significantly reduced activation in patients as compared to control subjects for the anticipation of dyspnea.

#### Associations between brain activation and patient characteristics

Correlation analyses in the patient group revealed that neural activation during dyspnea anticipation [cue severe vs. cue mild dyspnea] in the left hippocampus was negatively correlated with 6MWD and positively correlated with mMRC and HADS anxiety scores. Moreover, peak activations within the right amygdala and right hippocampus were positively correlated with reduced quality of life (i.e., higher SGRQ scores, Figure 3).

**Figure 4:**
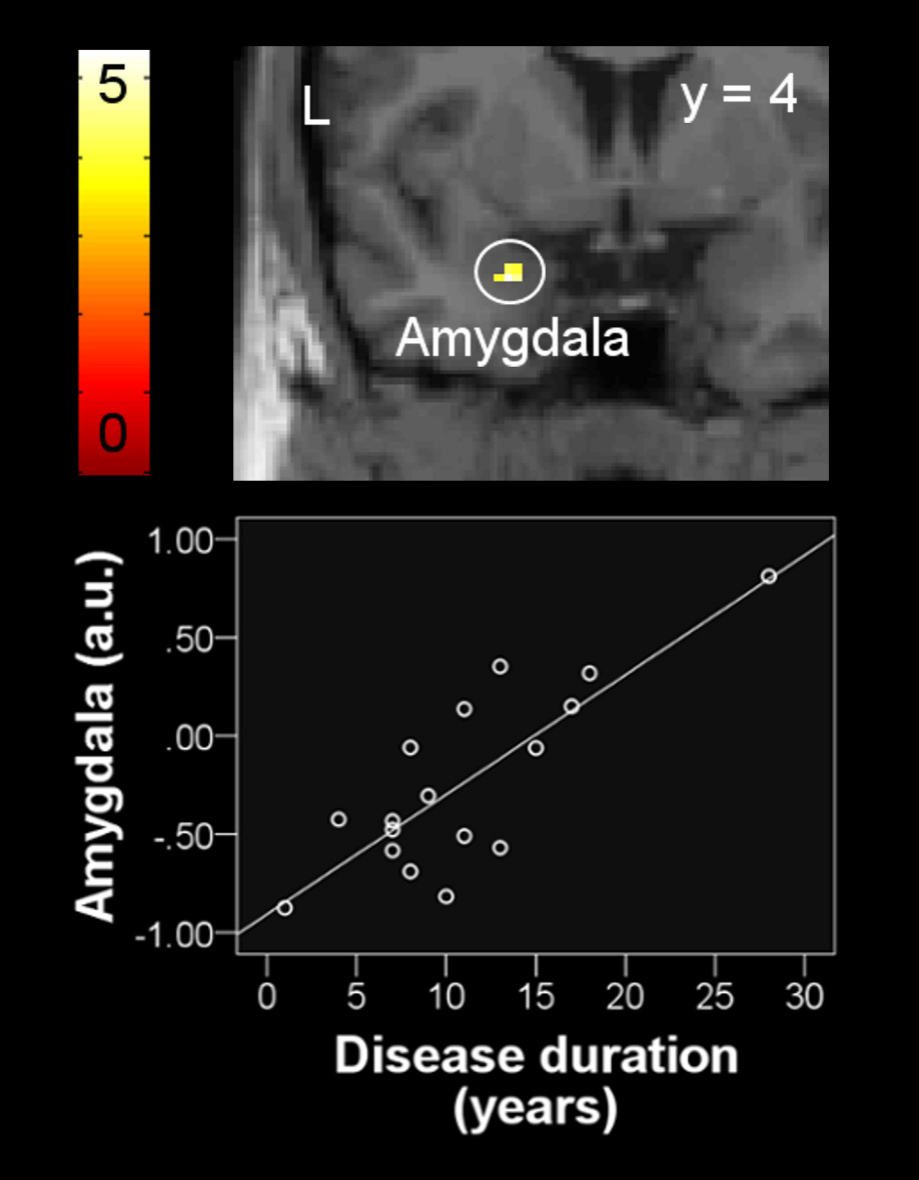
Positive correlation between left amygdala activation during the perception of increased dyspnea and disease duration in patients with COPD. For visual purposes, activation is thresholded at *p*_uncorrected_ < 0.001 with colorbars indicating *T*-values. Beta weights (y-axis) of individual subjects' peak voxel used in the scatter plot indicate neural activation using arbitrary units.

The lack of differences in brain activation between patient and control group during the perception of increased dyspnea allowed no offline partial correlation analyses for this contrast. Instead, explorative *post-hoc* analyses included the respective patient characteristics (e.g., disease duration, 6MWD, and scores for SGRQ, mMRC, and HADS anxiety and depression) as covariates-of-interest within a one-sample model for the patient group. These analyses revealed a significant positive correlation of disease duration with neural activation during increased dyspnea [severe vs. mild dyspnea] in the left amygdala (peak: x, y, z = −24, 2, −24, Z = 3.44, ROI-corrected *p* = 0.026, Figure 4).

## Discussion

The present fMRI study compared brain activation during the perception and anticipation of resistive-load induced dyspnea between patients with COPD and matched healthy controls. Furthermore, we investigated the relationship between COPD-specific brain activation and patient characteristics.

During the perception of increased dyspnea, patients and control subjects showed widely comparable neural activation patterns in commonly observed dyspnea-relevant sensorimotor and cortico-limbic brain regions without significant differences between groups. In contrast, while anticipating increased dyspnea, enhanced neural activation was observed in patients relative to control subjects in the bilateral hippocampus and right amygdala. Notably, neural responses in these emotion-related limbic brain regions were correlated with clinical patient characteristics. Specifically, patients with higher levels of anxiety and dyspnea as well as lower exercise capacity showed higher anticipatory neural activation in the left hippocampus, whereas patients with lower health-related quality of life showed higher anticipatory neural activity in the right hippocampus and amygdala. Additional explorative findings indicated that patients with longer disease duration showed higher amygdala activation during the perception of increasing dyspnea. Taken together, these results suggest that patients with stable moderate-to-severe COPD show enhanced activation in emotion-related brain areas than healthy control subjects during the anticipation, but not during the perception of experimentally induced increased dyspnea, which further relates to important clinical characteristics.

The observed brain activation in sensorimotor (SM1, SII/operculum, SMA, and thalamus) and cortico-limbic (ACC, insula, and PFC) areas during the perception of increased dyspnea is in line with previous neuroimaging studies in healthy volunteers (Peiffer et al., 2001; Evans et al., 2002; McKay et al., 2003; von Leupoldt et al., 2008; Pattinson et al., 2009; Binks et al., 2014). Our findings extend the notion of a dual cortical pathway model of dyspnea perception with pathways subserving either sensorimotor or affective aspects of dyspnea to patients suffering from COPD (von Leupoldt and Dahme, 2005; Davenport and Vovk, 2009). Moreover, the present finding that patients and control subjects demonstrated activation in comparable dyspnea-relevant brain regions corresponds with a recent study by Higashimoto et al. using near-infrared spectroscopy (Higashimoto et al., 2015). Although limited in spatial resolution, this study also demonstrated comparable activations in pre-motoric areas between COPD patients and healthy controls. In addition, several studies on chronic pain syndromes similarly observed substantial overlap of brain activation during the perception of acute pain in healthy subjects and patients with several pain conditions (Derbyshire et al., 2002; Apkarian et al., 2005, 2011; Baliki et al., 2006). However, the absence of enhanced responses in emotion-related areas during perceived dyspnea in COPD-patients is contrasting with previous studies on chronic pain conditions such as fibromyalgia, irritable bowel syndrome, and back pain (Baliki et al., 2006; Jensen et al., 2009; Hashmi et al., 2013). For example, Hashmi and colleagues (Hashmi et al., 2013) demonstrated a shift toward more activation in emotion related areas in those patients suffering from lower back pain, who developed chronicity. The lack of comparable changes in our study might be related to differences between studies regarding sensory modalities (e.g., thermal pain vs. load induced dyspnea), experimental designs (with vs. without anticipation conditions; different control conditions), disease duration, or patient characteristics (see below) including different exacerbations phenotypes of COPD (Scioscia et al., 2017), which deserves further investigation.

Importantly, during the anticipation of increased dyspnea, patients with COPD compared to healthy control subjects showed enhanced neural activation in hippocampus and amygdala, both key areas in the processing of fear, aversive learning, and pain (LeDoux, 2003; Sehlmeyer et al., 2009; Wiech and Tracey, 2013). Recent findings in healthy individuals demonstrated activation of the amygdala during the fearful anticipation of resistive load induced dyspnea (Stoeckel et al., 2015). Similarly, Apkarian et al. (2011) reported increased amygdala activation prior to a pain stimulus peak in healthy subjects, indicating a role of the amygdala in the anticipation of impending pain. The importance of amygdala and hippocampus in pain-related fear acquisition and memory processes was furthermore confirmed for patients with chronic irritable bowel syndrome (Icenhour et al., 2015). According to the aforementioned model regarding brain circuitry involved in the transition from acute to chronic pain (Apkarian et al., 2011; Mansour et al., 2014), the hippocampus and the amygdala are key regions of the limbic circuitry crucially involved in emotional enhancement processes. This limbic circuitry is presumed to “translate” sensory signals into more emotional, cognitive suffering states, e.g., by modulating and amplifying the emotional character of aversive nociceptive input signals over time (Mansour et al., 2014). Our finding of prominent recruitment of hippocampus and amygdala in COPD patients during the anticipation of increased dyspnea most likely reflects such an amplified emotional, in particular more fearful, evaluation of upcoming dyspnea. This might provide, at least partly, the neural basis for subsequent fearful avoidance behavior in patients with COPD and contribute to a spiral of decline in form of activity avoidance, deconditioning, increased dyspnea and anxiety, and further reductions in quality of life (Reardon et al., 2006; Troosters et al., 2013). Support for this assumption comes from the present observation that increased activation of hippocampus and amygdala during increased dyspnea anticipation was closely related to important clinical characteristics such as reduced exercise capacity and quality of life as well as higher levels of dyspnea and anxiety. Notably, using structural MRI, morphological changes of reduced gray matter volume in hippocampus and amygdala were recently observed in patients with COPD relative to control subjects (Esser et al., 2016), which further supports the important role of these areas in COPD.

Finally, *post-hoc* analyses revealed a positive correlation between disease duration and amygdala activation during the perception of increased dyspnea. Although explorative in nature, this finding suggests that the chronic course of COPD results in a functional modulation of a brain region strongly related to fear and anxiety (LeDoux, 2003; Sehlmeyer et al., 2009; Wiech and Tracey, 2013). Interestingly, a recent study showed that longer COPD disease duration was also related to reduced gray matter volume in the ACC (Esser et al., 2016), a key structure for antinociception and the regulation of emotional states that is tightly interconnected with the amygdala (Tracey and Mantyh, 2007). Given the fact that many patients with COPD develop comorbid anxiety over time (Maurer et al., 2008; Yohannes and Alexopoulos, 2014), it might be speculated that this is related to increased amygdala activation during dyspnea anticipation and perception, paralleled by reduced antinociceptive ACC capabilities. However, future studies are required directly addressing this assumption.

When interpreting the present results, several limitations need to be taken into account. We exclusively studied a small group of highly motivated patients with stable health status and relatively good physical condition without clinical levels of comorbid anxiety and depression and no exacerbation history within the last year. This limits the generalizability of our findings and motivates future studies in other patient populations with larger sample sizes, different and/or more severe forms of COPD and related comorbidities. Moreover, the use of resistive-load breathing for the experimental induction of short-lasting dyspnea only mirrors some aspects of dyspnea, i.e., the sense of increased work and effort of breathing, and might not be comparable to sustained dyspnea experiences outside the lab. Therefore, future studies are necessary to extend the present findings to other qualities of dyspnea, for example air hunger, which might be perceived and/or anticipated differently (Banzett et al., 2008). Finally, future longitudinal studies are needed to directly address how changes in the neural processing of perceived and anticipated dyspnea relate to the subsequent development of activity avoidance, deconditioning, increased dyspnea and anxiety, and reduced quality of life.

This study suggests that patients with stable moderate-to-severe COPD show enhanced activation in emotion-related brain areas compared with healthy control subjects during the anticipation, but not during the perception, of increased resistive-load induced dyspnea. Brain activation in these areas was related to important clinical characteristics and might contribute to the development of a downward spiral including fearful activity avoidance, deconditioning, increased dyspnea and anxiety, and reduced quality of life. Taken together, our findings contribute to our understanding of brain processes in COPD and their relation with clinical outcomes and might provide potential targets for future psychological interventions aimed at improving the burden for patients.

## Author contributions

RE, MS, HM, and AV contributed to the conception and study design; RE, MS, AK, HW, KT, and KL contributed to the data acquisition; RE, MS, CB, and AV contributed to the data analysis; RE, MS, and AV drafted the paper; all authors contributed to the interpretation of data, the editing of the paper, provided critical revisions and approved the final version of the manuscript.

### Conflict of interest statement

KT and KL were employed by company Atem-Reha GmbH. The authors declare that research was conducted in the absence of any commercial or financial relationship that could be construed as a potential conflict of interest. The other authors declare that the research was conducted in the absence of any commercial or financial relationships that could be construed as a potential conflict of interest.
