# Supplementary material for: Brain Activation during Perception and Anticipation of Dyspnea in Chronic Obstructive Pulmonary Disease
Source: Front Physiol. 2017 Aug 23;8:617. doi: 10.3389/fphys.2017.00617 (PMC5572159; doi:10.3389/fphys.2017.00617)
Supplement: Supplementary file 1 [file DataSheet1.pdf]

Brain Activation during Perception and Anticipation of Dyspnea in Chronic Obstructive Pulmonary Disease

Roland W. Esser [1], Maria C. Stoeckel [1], Anne Kirsten [2], Henrik Watz [2], Karin Taube [3], Kirsten Lehmann [3], Helgo Magnussen [2], Christian Büchel [1] and Andreas von Leupoldt [1,4*]

Supplementary data sheet

Results

Respiratory Parameters

For both groups, P_I and T_I were significantly higher (severe vs. mild load: P_I, $p < .001$ for both groups; T_I, $p < .01$ for COPD and $p < .05$ for control group), while V_T and V_E were significantly lower (severe vs. mild load: V_T, $p < .01$ for COPD and $p < .05$ for control group; V_E, $p < .001$ for both groups) during the severe than during the mild dyspnea condition. No significant differences in respiratory parameters were observed between anticipation of mild and severe dyspnea for both groups, except for V_E. V_E values were higher during mild than severe dyspnea anticipation in both groups, but the difference between conditions was found to be significant only in the patient group (cue severe dyspnea vs. cue mild dyspnea, $p < .001$). PET_{CO2} and f were constant across all experimental conditions for both groups. Although patients compared to control subjects demonstrated some differences in f, V_E, and T_I throughout experimental conditions, the difference in all respiratory parameters between the mild and severe dyspnea condition (severe dyspnea minus mild dyspnea) and anticipation of mild and severe dyspnea (cue severe dyspnea minus cue mild dyspnea) were comparable between groups. This suggests similar changes in respiratory patterns from mild to severe dyspnea and from anticipation of mild dyspnea to anticipation of severe dyspnea in both patients and controls. In both groups, PET_{CO2} levels were relatively low across all conditions. This mildly hypocapnic pattern might have resulted from face mask breathing in a supine position within the narrow scanner environment. However, PET_{CO2} levels were comparable across all experimental conditions and groups and were controlled for in each subject by the inclusion as covariate-of-no-interest in the statistical models, which renders a potential influence on the fMRI data rather unlikely.

Table S1 | Group means (SD) for respiratory parameters during experimental conditions for patients with COPD and control subjects.

	COPD Dyspnea				Controls Dyspnea			
	Anticipation		Perception		Anticipation		Perception	
	mild	severe	mild	severe	mild	severe	mild	severe
PET_{CO2}, mmHg	28.82 (4.51)	28.63 (4.49)	29.17 (3.95)	28.9 (4.0)	30.96 (4.93)	30.5 (4.58)	30.56 (4.47)	30.63 (4.61)
P_I, mbar	1.66 (1.12)	1.37 (.26)	2.56 (1.0)	10.04 (4.87)***	1.29 (.41)	1.47 (1.66)	2.4 (.7)	8.49 (3.59)***
V_T, L	.8 (.25)	.77 (.27)	.97 (.27)	.88 (.31)**	.82 (.21)	.79 (.23)	1.11 (.32)	.98 (.35)*
f, breaths/min	18.91 (6.29)	18.54 (6.48)	15.74 (4.0)	15.87 (4.15)	12.98 (4.12) †††	13.21 (3.93) ††	12.92 (4.25) †	12.34 (4.43) †
V_E, L/min	13.63 (4.33)	12.53 (3.84)***	14.45 (3.96)	12.74 (3.54)***	10.08 (3.47) ††	9.41 (1.96) ††	13.28 (3.33)	10.73 (3.39)***
T_I, s	1.58 (.81)	1.59 (.73)	1.7 (.47)	1.88 (.59)**	2.41 (.86) ††	2.28 (.78) ††	2.29 (.72) ††	2.56 (.93)* †

Abbreviations: PET_{CO2} = Partial pressure of end-tidal CO_2; P_I = Peak inspiratory pressure; V_T = Tidal volume; f = Breathing frequency; VE = Minute ventilation; TI = Inspiratory time.

*p < .05, **p < .01, ***p < .001 for respective comparisons between mild and severe dyspnea periods separately calculated for the anticipation and perception within each group.

†p < .05, ††p < .01, †††p < .001 for between group comparisons calculated separately for each condition (mild anticipation, severe anticipation, mild perception, and severe perception).

Table S2 | MNI-space peak coordinates, z-values, and p-values for regions showing significant brain activation during increasing dyspnea perception in control subjects.

Brain Region	x	y	z	Z	*p*
L SM1	-48	-16	34	5.43	.003[†]
R	54	-20	32	6.01	< .001[†]
L SII/operculum	-42	-28	14	5.22	.008[†]
R	64	-6	12	5.63	.001[†]
R SMA	8	0	54	4.76	.001[*]
L Thalamus	-20	-20	8	4.97	.012[†]
R	18	-16	12	4.52	.002[*]
L Insular Cortex					
- Posterior	-36	-8	2	4.97	.022[†]
- Anterior	-32	24	-8	3.85	.036[*]
R	42	8	0	4.89	.031[†]
L PFC (dorso-medial)	-4	20	42	3.91	.033[*]

Abbreviations: L= left hemisphere; R = right hemisphere; SM1 = primary sensorimotor cortex; SII = secondary somatosensory cortex; SMA = supplementary-motor area; PFC = prefrontal cortex.
[†]whole-brain family-wise error corrected, [*]corrected for multiple comparisons within respective bilateral ROIs.

Table S3 | MNI-space peak coordinates, z-values, and p-values for regions showing shared significant brain activation in the conjunction analysis between the patient and control group during increasing dyspnea perception.

Brain Region	x	y	z	Z	*p*
L SM1	-50	-16	34	6.25	< .001[†]
R	60	-8	34	4.91	.019[†]
L SII/operculum	-56	-6	10	4.96	.016[†]
R	52	10	-2	4.70	.001[*]
R SMA	8	-2	56	4.84	.027[†]
L Thalamus	-18	-20	8	4.68	.001[*]
R	16	-18	8	3.55	.041[*]
L Insular Cortex					
- Posterior	-36	-8	2	4.81	.030[†]
- Anterior	-36	22	-4	3.79	.038[*]
R	50	10	-2	4.60	.002[*]
R PFC (dorso-medial)	2	18	42	4.07	.017[*]

Abbreviations: L= left hemisphere; R = right hemisphere; SM1 = primary sensorimotor cortex; SII = secondary somatosensory cortex; SMA = supplementary-motor area; PFC = prefrontal cortex.

[†]whole-brain family-wise error corrected, [*]corrected for multiple comparisons within respective bilateral ROIs.
